# Detection of unsafety in families with parental and/or child developmental problems at the start of family support

**DOI:** 10.1186/s12888-016-0715-y

**Published:** 2016-01-21

**Authors:** Claudia E. van der Put, Jo Hermanns, Loes van Rijn-van Gelderen, Frouke Sondeijker

**Affiliations:** Research Institute of Child Development and Education, University of Amsterdam, Nieuwe Achtergracht 127, Amsterdam, 1018 WS The Netherlands; H&S Consult, Leidsestraatweg 133, Woerden, 3443BT The Netherlands; The opvoedpoli, Houtmankade 332, Amsterdam, 1013RR The Netherlands

**Keywords:** Risk assessment, Child maltreatment, California Family Risk Assessment (CFRA)

## Abstract

**Background:**

Risk assessment is crucial in preventing child maltreatment as it can identify high-risk cases in need of child protection intervention. Despite this importance, there have been no validated risk assessment instruments available in the Netherlands for assessing the risk of child maltreatment. Therefore, the predictive validity of the California Family Risk Assessment (CFRA) was examined in Dutch families who received family support. In addition, the added value of a number of experimental items was examined. Finally, it was examined whether the predictive value of the instrument could be improved by modifying the scoring procedure.

**Methods:**

Dutch families who experienced parenting and/or child developmental problems and were referred by the Centres for Youth and Family for family support between July 2009 and March 2011 were included. This led to a sample of 491 families. The predictive validity of the CFRA and the added value of the experimental items were examined by calculating AUC values. A CHAID analysis was performed to examine whether the scoring procedure could be improved.

**Results:**

About half of the individual CFRA items were not related to future reports of child maltreatment. The predictive validity of the CFRA in predicting future reports of child maltreatment was found to be modest (AUC = .693). The addition of some of the experimental items and the modification of the scoring procedure by including only items that were significantly associated with future maltreatment reports resulted in a ‘high’ predictive validity (AUC = .795).

**Conclusions:**

This new set of items might be a valuable instrument that also saves time because only variables that uniquely contribute to the prediction of future reports of child maltreatment are included. Furthermore, items that are perceived as difficult to assess by professionals, such as parental mental health problems or parents’ history of abuse/neglect, could be omitted without compromising predictive validity. However, it is important to examine the psychometric properties of this new set of items in a new dataset.

**Electronic supplementary material:**

The online version of this article (doi:10.1186/s12888-016-0715-y) contains supplementary material, which is available to authorized users.

## Background

Child maltreatment is a serious problem with consequences for individual children and society. Child maltreatment may include physical abuse, sexual abuse, emotional abuse and neglect (including witnessing domestic violence). In the Netherlands, where the current study was conducted, the annual prevalence rate is estimated to be 34 victims per 1,000 children based on official reports and 99 victims per 1,000 children based on self-reports [1]. Approximately half of these cases involve serious child maltreatment [1]. The number of children that die as a result of child abuse and neglect in the Netherlands is not known but is estimated to be in double figures, up to about 50 per year [2]. In a considerable number of these cases, child welfare workers were involved in one way or another [3], which raises the question whether safety risks were sufficiently addressed.

Worldwide, the development and evaluation of risk assessment instruments in the field of child protection is still in its infancy. Thus far, there are two major approaches to risk assessment in child protection: actuarial and clinical judgment. The main difference is that, in an actuarial approach, conclusions are based solely on empirically established relationships between data and the outcome of interest, whereas in a clinical approach, conclusions are based on the judgment of a professional who combines and weighs information in a non-standardized manner [4]. Clinical approaches can be further divided into: (a) unaided decision making based on experience, knowledge and intuition (unstructured clinical judgment), (b) tools based on opinions of experts but without empirical bases (consensus-based instruments), and (c) tools with empirical basis that leave the final decision-making process to the professional (structured clinical judgment [SCJ]).

International validation studies indicate that the performance of most clinical methods, including unstructured clinical judgment and consensus-based methods, is questionable [5–16]. Furthermore, studies comparing different methods have consistently shown that actuarial instruments outperform both unstructured and consensus-based instruments at estimating risks within different domains, such as child welfare, criminal justice, forensic mental health, and clinical psychology [4, 8, 10, 17, 18]. In addition, research indicates that actuarial methods are better than [19] or equally effective as [20] SCJ instruments in predicting violent behavior. One of the explanations for the superior predictive performance of actuarial methods is that the reliability of actuarial instruments is higher, because the scoring and combining of risk factors occurs according to a fixed algorithm, meaning that professionals use the same scoring rules, whereas in clinical methods the scoring and combining of risk factors is done in a subjective fashion [4]. Despite these results, clinical approaches are more common in child protection practices than actuarial instruments, both internationally and in the Netherlands.

In the Netherlands, no validated instruments are available for assessing the risk of child maltreatment. The instruments currently in use in Dutch child protection services are the Light Instrument Appraisal Child Maltreatment (LIRIK [21]), the Child Abuse Risk Evaluation-Netherlands (CARE-NL [22]), and the Delta Safety List [23]. These instruments are all clinical based and differ slightly in their aims: The CARE-NL is used to examine the risk of child maltreatment after a child is referred to the Dutch Advice and Reporting Center of Child Abuse (ARCAN); the LIRIK is used for screening the risk of child maltreatment at the start of family support by the Dutch Youth Care Agency (BJZ); and the aim of the Delta Safety List is to monitor safety during a child protection intervention. Unfortunately, these instruments were implemented without any (Dutch or international) validation research and therefore it is not known how well these instruments actually perform [24, 25].

As abovementioned, the instruments currently in use in the Dutch child protection services to assess risks of child maltreatment are clinical-based. Because implementing actuarial methods during risk assessment in child protection services may be promising [4, 8, 10, 17, 18], an actuarial instrument (the California Family Risk Assessment [CFRA]) was introduced in a regional pilot to be used by family coaches and intervention nurses of the Dutch Center for Youth and Family. The CFRA is an actuarial risk assessment instrument originally designed for assessing the risk of child maltreatment subsequent to receipt of an initial maltreatment report [26, 27]. The only study to our knowledge that tested the predictive validity of the CFRA was performed in California and found an AUC value of .63 [27]. Although this means that the CFRA did not perform well in California, we chose this instrument because the CFRA was evaluated as short, user-friendly and easy to adapt to the Dutch situation. In addition, we were not familiar with instruments suitable for Dutch practice that outperformed the CFRA when applied in every-day practice. Comparing the performance of the CFRA with the average performance of other instruments for child maltreatment is not possible because no meta-analyses have been conducted in this field and therefore the average AUC of risk assessment instruments for child maltreatment is still unknown. Until research identifies actuarial models exhibiting superior predictive validity when applied in every-day practice, the CFRA is a useful instrument [27]. Also, in the field of youth delinquency, several meta-analyses on the performance of risk assessment instruments have been conducted [28–30] and the AUC value of the CFRA is approximately equal to the mean AUC value of instruments frequently used in forensic settings.

To examine whether the AUC value of the CFRA could be improved in the future, the added value of a number of experimental items was examined. The experimental items were not taken into account in the calculation of the CFRA risk scores, so the CFRA was used as originally intended. The experimental items were chosen for the following three reasons. Firstly, the CFRA is implemented in the Netherlands slightly different than in California. In California the CFRA is used for assessing risks *after* a maltreatment report, concerning families who are often not (yet) in treatment at child welfare agencies, whereas in the Netherlands the CFRA is used at the start of treatment, mostly *without* a prior maltreatment report. In treating these families, professionals often experience resistance to treatment and poor motivation to participate in treatment. Therefore, experimental items have been added to measure motivation to change and adherence to therapy. As far as we know, there is no strong empirical evidence for the relationship between these items and future child maltreatment: however, we have added these items as experimental items on the basis of professionals’ practical experiences.

Secondly, child maltreatment research has generally focused on mothers, although mothers and fathers perpetrate child maltreatment at similar rates [31]. Possible explanations for the underrepresentation of fathers compared to mothers in child maltreatment research include the idea that researchers perceive mothers as being more important than fathers in the nurturing and development of children and the increased accessibility of mothers for study recruitment [32]. In the CFRA, most items are about the primary caretaker, defined as the parent living in the household who assumes the most responsibility for childcare, who is the mother in most cases. To gain more knowledge about the importance of the risk factors concerning fathers, experimental items have been added also regarding risk factors for the secondary caretaker. The secondary caretaker is defined as an adult living in the household (in most cases the father) who has routine responsibility for childcare, but less responsibility than the primary caretaker.

Finally, some experimental items are added because previous studies have shown that they are associated with future child maltreatment, such as: primary caretaker lacks pedagogical skills, primary caretaker has low self-esteem and the family has financial problems [33, 34].

To summarize, the aims of the present study were to examine the predictive validity of the CFRA in the Netherlands in families with parenting and/or child developmental problems; to test the added value of the experimental items on the predictive validity; and to examine whether the predictive value can be improved by modifying the scoring procedure. The following research questions were addressed: (1) What is the strength of the relationship between the individual items of the CFRA and future reports of child maltreatment, (2) What is the strength of the relationship between the experimental items and future reports of child maltreatment, (3) What is the internal consistency and predictive validity of the CFRA in the Netherlands, (4) To what extent can the predictive validity of the CFRA be improved by adding experimental items, and (5) To what extent can the predictive validity be improved by using a different scoring procedure?

## Methods

### Sample

In the Netherlands, families are routinely seen in the Centres for Youth and Family [Centra voor Jeugd en Gezin] for health and developmental check-ups by the preventive child and youth health care services. Families can also voluntarily visit the public health nurses in the Centres for Youth and Family or be referred to them by other professionals. Our sample consisted of 491 families that, after a first analyses of the family needs by the public health nurses of the Centres for Youth and Family in the city of Rotterdam, were referred to specialized and more extensive family support in the period from July 2009 to March 2011. All families had at least one child aged 0-12 years and were referred because of parenting and/or child developmental problems.

Dutch intervention nurses offer short intensive counseling and make home visits to hard-to-reach families with parenting and/or child developmental problems in order to introduce families to other services, whereas family coaches offer an intensive counseling program for families facing multiple problems, make a plan of action and coordinate assistance and referrals to youth health care and welfare agencies. Table 1 presents the background characteristics of the families in the sample.

**Table 1:** Sample characteristics (*N* = 491)

	Number	Percent
Ethnicity		
Dutch	165	33.6 %
Surinamese, Antillean	96	19.6 %
Moroccan, Turkish	105	21.4 %
Other (e.g., Cape Verdeans, other Africans, and Eastern Europeans)	125	25.5 %
Age primary caretaker		
Older than 30	260	53.0
30 or younger	231	47.0
Family size		
Number of children	M = 2.05 (SD = 1.15)	
Number of adults	M = 1.70 (SD = .78)	

*Note*: M = mean, SD = standard deviation

### Procedure

The CFRA was evaluated as short, user-friendly and easy to adapt to the Dutch situation. A pilot study was conducted in collaboration with the Centers for Youth and Family. The objective of the present study was not the reason to introduce the CFRA at the Centers for Youth and Family. The CFRA was completed by family coaches and intervention nurses from the Center for Youth and Family Rotterdam Rijnmond. The family coaches and intervention nurses received a training consisting of two sessions in which they learned how to use the CFRA. Specifically, the training included the following elements: (a) an explanation of the purpose and use of the instrument, (b) lessons on how to identify risk factors, (c) completing the instrument on the basis of their own case studies, and (d) discussing issues, questions and ambiguities encountered in completing the CFRA. Minor adjustments were made to both the instrument and the instruction to clarify certain ambiguities on the basis of the comments received during training. Subsequently, family coaches practiced the use of the CFRA during an exercise period of two months. The copies of the CFRA that were completed during this period are not included in the analyses. Intervention nurses have not practiced completing the CFRA after receiving training, because they were involved in the project later on due to a low caseload of family coaches. The data collection took place from July 2009 until March 2011 and led to 491 completed CFRAs.

### Instruments

#### The California Family Risk Assessment (CFRA)

The CFRA is an actuarial risk assessment instrument for assessing the risk of child maltreatment. This instrument was developed in 1998 by the Children’s Research Center, a unit of the National Council on Crime and Delinquency, as part of California’s child welfare structured decision-making (SDM) model [27]. The CFRA consists of 20 case attributes divided into two 10-item scales, one scale to assess the likelihood of future abuse (physical or sexual), and another to assess the likelihood of future neglect. The total risk score for *neglect* is calculated by summing the items assessing the likelihood of future neglect, and the total risk score for *abuse* is calculated by summing the items assessing the likelihood of future abuse (see Additional file 1 for items assessing the risk of future abuse, response options, and risk scores). The wording of some of the items of the CFRA was adapted slightly to the purpose for which the CFRA is used in the Netherlands: “current complaint is for neglect” was changed into “current intervention is aimed at neglect”, “current complaint is for abuse” was changed into “current intervention is aimed at abuse” and “prior investigations” was changed into “prior interventions, investigations, reports”.

The total risk score for *each case* is calculated by applying cut-off scores, for both the total risk score for neglect and the total risk score for abuse, to produce ratings of low, moderate, high, or very-high-risk (see Additional file 1). The highest risk score produced by either scale is taken as the total risk score of the CFRA [27].

Alongside this procedure, the CFRA provides the opportunity to override the calculated risk scores. Child welfare workers are allowed to override the risk assessment scores obtained with the standard CFRA scoring procedure and to increase the risk ratings under two conditions ([27], p 19): “(1) a one-category risk score increase is allowed with supervisory concurrence when the child welfare worker’s clinical impressions suggest that the case is higher-risk than the standard CFRA score indicates, and (2) any risk score below very-high risk can be changed to very-high risk in the presence of case attributes agreed by program administrators to indicate greater risk to children”.

#### Experimental items

As abovementioned, a number of experimental items were used in the present study with the aim to examine whether the predictive value of the CFRA could be improved in the future by adding these items. The experimental items were not taken into account in the calculation of the CFRA risk scores, so the CFRA was used as originally intended. Table 2 shows the experimental items, response options, and risk scores.

**Table 2:** Experimental items that were added to the CFRA

1.	Primary caretaker kept the child from school/allowed the child to illegally not attend school (in the past year)	Never	0
		Once	2
		More than once	2
2.	The child was found unattended on the streets (in the past year)	Never	0
		Once	2
		More than once	2
3.	The child was admitted to hospital urgently/ taken to the emergency room	Never or once	1
		Twice	2
		More than twice	2
4.	The family has financial problems	No	0
		Yes	1
7.	Primary caretaker characteristics	No problems	0
		Lacks pedagogical skills	1
		Low self-esteem	1
		Apathetic or desperate	1
8.	Primary caretaker is involved in destructive relationships	No	0
		Yes, but not as a victim of domestic violence	1
		Yes, as a victim of domestic violence	2
9.	Activities to improve parenting skills	Performs all agreed actions	0
		Performs only some agreed actions	1
		Performs practically no agreed actions	2
10.	Participation in the intervention: primary caretaker cancels appointments/is not present at appointments	Never without a good reason	0
		Once	1
		More than once	2
11.	Primary caretaker believes that the parenting problems are less severe than indicated by the professional	No	0
		Yes	1
12.	Secondary caretaker characteristics	No problems	0
		Provides insufficient emotional/psychological support	1
		Is overly or for incomprehensible reasons strict with the child	1
		Is very dominant	1
13.	Secondary caretaker was abused or neglected in his youth	No	0
		Yes	1
14.	Secondary caretaker has previous alcohol and/or drug problems	No	0
		Yes	1
15.	Secondary caretakers has alcohol and/or drug problems at the present	No	0
		Yes	1
16.	Activities to improve parenting skills (secondary caretaker)	Performs all agreed actions	0
		Performs only some agreed actions	1
		Performs practically no agreed actions	2
17.	Participation in the intervention: secondary caretaker cancels appointments/is not present at appointments	Never without a good reason	0
		Once	1
		More than once	2

#### Outcome measure

Existing data were used for the outcome measure. Reports (yes/no) of child maltreatment in the family at the Dutch Advice and Reporting center of Child Abuse and Neglect (ARCAN) during a follow-up period of 6 months *after* completing the CFRA. Both verified and unverified reports were included. Reason for this was that the process of verification often takes longer than the time window of the study.

There are twelve ARCANs in the Netherlands. Professionals and non-professionals can call upon these services for advice and/or to report suspicions of child abuse. They receive advice about their potential (active) role and options, or they can formally report a suspicion of child maltreatment. After investigation of the report there are three main routes: 1) to arrange access to care (youth care, mental healthcare, social work, and parent support), 2) to provide protection, or 3) to report to the police and/or prosecutor.

### Analyses

First, the relationships between the individual items of the CFRA and future reports of child maltreatment were examined by calculating phi coefficients ϕ.

Second, the relationships between the *experimental* items and future reports of child maltreatment were examined by calculating phi coefficients ϕ.

Third, the predictive validity of the CFRA was assessed by calculating the Area Under the receiver operating characteristic Curve (AUC) value. The AUC indicates what percentage of correct classifications the instrument will yield overall [35]. When the AUC is 0.50, it indicates that the instrument is not better than making predictions by chance. A value of 1.00 indicates a perfect positive prediction and a value of 0.00 indicates a perfect negative prediction. According to generally accepted criteria, AUC values of 0.70 and above are acceptable, and values of 0.75 are considered good.

Fourth, the internal consistency for the two scales of the CFRA (the scale for assessing the likelihood of future neglect and the scale for assessing the likelihood of future abuse) was examined by calculating Cronbach’s alphas.

Fifth, the added value of the experimental items was examined by testing the significance of the difference between (a) the AUC value of the sum of CFRA items that were significantly associated with future reports of child maltreatment and (b) the AUC value of the sum of CFRA and experimental items that were significantly associated with future reports of child maltreatment. To test whether the AUC values differed significantly, we used the Hanley and McNeil method [36].

Sixth, to examine whether the predictive validity could further be improved, a Chi-squared Automatic Interaction Detector (CHAID) analysis was performed. CHAID is a classification tree method, which focuses on interactions rather than on main effects in the data set being examined. In the first step of the CHAID procedure, the total group of subjects is divided into a number of subgroups on the basis of the variable most strongly associated with the outcome. In the second step, the groups are split again on the basis of the variable that is then most strongly associated with child maltreatment. This procedure is repeated until no variables remain that have a significant association with child maltreatment in the subgroups, or until the groups have reached a minimum size. In the present study, a minimum value of *n* = 20 was used. Input variables for the CHAID analyses were the individual CFRA items, the experimental items and the sum scores (sum of significant CFRA items, sum of significant CFRA and experimental items). CHAID is highly appropriate for gaining insight into family profiles with a high and low risk of child maltreatment, respectively, because it identifies groups of cases that share the same risk factors and also share the same value or risk [36, 37]. Another advantage of CHAID is that the results are visual and therefore easy to interpret.

Finally, the sensitivity, specificity, percentages of false positives, and percentages of false negatives at various cut-off scores were examined. In addition, Youden’s index was calculated. Youden’s index (*J*) is Sensitivity + Specificity -1 [38]. Youden’s index can be used to examine the optimal cut-off with maximal sensitivity and specificity. The index ranges from 0 to 1, and has a zero value when a diagnostic test gives the same proportion of positive results for groups with and without the outcome of interest, i.e. the test is useless. A value of 1 indicates that there are no false positives or false negatives, i.e. the test is perfect. Sensitivity is the probability of a positive score on the CFRA for children who will actually be reported for child maltreatment; specificity is the probability of a negative score on the CFRA for children who will not be reported for child maltreatment. A false negative is a negative score on the CFRA while in reality the child is at risk of child maltreatment. A false positive is a positive score on the CFRA while in reality the child is not at risk of maltreatment.

### Ethical approval

Formal Institutional Review Board (IRB) approval to conduct this study was not required, nor was informed consent of the participants, as this study involved secondary data analysis on de-identified data, which does not impose any harm on the subjects and does therefore not necessitate IRB regulation. Accordingly, this study was conducted ethically based on the rules maintained by the Faculty Ethics Review Board (FMG-UvA) of the University of Amsterdam, The Netherlands. Permission to use the de-identified data was provided by Dr. O. de Zwart, Director of the Division of Youth, Department of Youth and Education of the City of Rotterdam.

## Results

### Relationship between individual items of the CFRA and future reports of maltreatment

Table 3 shows the phi coefficients (ϕ) between the individual items of the CFRA and future reports of maltreatment. In 21 of the families (4.3 %), there were reports of child maltreatment in the follow-up period. Only four items of the 10-items scale to assess the likelihood of future neglect were related to future reports of maltreatment, namely: ‘current intervention is for neglect’, ‘prior neglect investigations’, ‘primary caretaker provides physical care inconsistent with child’s needs’, and ‘primary caretaker has a current alcohol or drug problem’. Almost half of the items assessing future *abuse* were significantly related to future reports of child maltreatment. These items were ‘current intervention is for abuse’, ‘prior abuse investigations’, ‘domestic violence in the household in the past year’, ‘primary caretaker is overly strict with the child’, and ‘primary caretaker is very dominant’.

**Table 3:** Phi-coefficients between CFRA items and future reports of child maltreatment

Item		ϕ
Items assessing future neglect		
1.	Current intervention is for neglect	.17^***^
2.	Prior neglect interventions, reports, investigations	.12^**^
3.	Child protection services received previously	-.05
4.	Number of children involved in incident	.03
5.	Age of youngest child in the home	-.02
6.	Primary caretaker provides physical care inconsistent with child’s needs	.11^*^
7.	Primary caretaker has past/current mental health problems	.06
8.a	Primary caretaker has a history of alcohol problems	.03
8.b	Primary caretaker has a history of drug problems	.07
8.c	Primary caretaker has a current alcohol problem	.06
8.d	Primary caretaker has a current drug problem	.16^***^
9.a	Characteristics of children: medically fragile/insufficient growth	-.03
9.b	Characteristics of children: developmental disorder or disabled	-.01
9.c	Characteristics of children: intoxicated at birth	-.01
10.a	Current housing is unsafe	.09^+^
10.b	No fixed place to live	-.01
Items assessing future abuse		
1.	Current intervention is for abuse	.16^***^
2.	Prior interventions for abuse, reports, investigations	.11^*^
3.	Previously received child protection services	-.03
4.	Prior injury to a child resulting from child abuse or neglect	.06
5.a	The fault lay with the child	.15^**^
5.b	The caretaker justified the abuse	-.01
6.	Domestic violence in the household in the past year	.13^**^
7.a	Primary caretaker gives insufficient emotional and psychological support	.01
7.b	Primary caretaker is overly strict with the child	.11^*^
7.c	Primary caretaker is very dominant	.24^***^
8.	Primary caretaker has a history of abuse or neglect as a child	.06
9.	Secondary caretaker has a previous or current alcohol or drug problem	-.05
10.a	Characteristics of children: delinquency	.04
10.b	Characteristics of children: developmental disorder/intellectual disability	.06
10.c	Characteristic of children: mental health problems or behavioral problems	.02

*Note.* ^*^ *p* < .05; ^**^ *p* < .01;^***^ *p* < .01

### Relationship between experimental items and future reports of maltreatment

Table 4 shows the phi coefficients (ϕ) between the experimental items and future reports of maltreatment. Five of the experimental items were related to future reports of maltreatment: ‘the family has financial problems’, ‘primary caretaker is involved in destructive relationships’, ‘extent to which primary caretaker performs activities to improve parenting skills’, and ‘extent to which primary caretaker cancels appointments/is not present at appointments’.

**Table 4:** Phi-coefficients between experimental items and Future Reports of Child Maltreatment

Item		ϕ
1.	Primary caretaker kept the child from school for illegal reasons	.06
2.	The child was found unattended on the streets	.07
3.	The child was admitted to hospital urgently/taken to emergency room	-.02
4.	The family has financial problems	.10^*^
5.a	Primary caretaker lacks pedagogical skills	.03
5.b	Primary caretaker has low self-esteem	-.03
5.c	Primary caretaker is apathetic or desperate	.05
6.	Primary caretaker is involved in destructive relationships	.11^*^
7.	Extent to which primary caretaker performs activities to improve parenting skills	.16^***^
8.	Extent to which primary caretaker cancels appointments/is not present at appointments	.16^***^
9.	Primary caretaker believes that the parenting problems are less severe than indicated by the professional	.22^***^
10.a	Secondary caretaker provides insufficient emotional/psychological support	-.06
10.b	Secondary caretaker is overly (or for inexplicable reasons) strict with the child	.09
10.c	Secondary caretaker is very dominant	.07
11.	Secondary caretaker was abused or neglected in his youth	.08
12.	Secondary caretaker has previous alcohol and/or drug problems	-.05
13.	Secondary caretaker has alcohol and/or drug problems at the present	-.04
14.	Extent to which secondary caretaker performs activities to improve parenting skills	-.04
15.	Extent to which secondary caretaker cancels appointments/is not present at appointments	.14

*Note.* ^*^ *p* < .05; ^***^ *p* < .01

### Predictive validity of the CFRA

Table 5 shows the point-biserial correlations (*r*_pb_) and the AUC values predicting future reports of child maltreatment for the neglect risk score (total score and categorized score), the abuse risk score (total score and categorized score) and the overall risk score (total and categorized score). The AUC values of the risk score for neglect, the risk score for abuse and the CFRA risk score were respectively .653 (.530-.776), .719 (.610 - .829) and .693 (.589 - .797).

**Table 5:** AUC values of the subscales and total risk score of CFRA (including categorization) in predicting future reports of child maltreatment

	AUC (95 % C.I.)
Neglect scale CFRA	
Total score	.669 (.537-.801)
Categorized score: low, moderate, high, very high	.653 (.530-.776)
Abuse scale CFRA	
Total score	.716 (.596-.836)
Categorized score (low, moderate, high, very high)	.719 (.610-.829)
Total risk score CFRA	
Total score (sum of risk score neglect and risk score abuse)	.719 (.603-.835)
Categorized score (low, moderate, high, very high)	.693 (.589-.797)

Cronbach’s alphas for the neglect and abuse scale were, respectively, .38 and .54. Cronbach’s alphas could be improved to .58 for the neglect scale and .57 for the abuse scale by deleting items based on the statistic “Cronbach’s alpha if item deleted”. Cronbach’s alpha was .61 for the total scale. Deleting the items ‘age of youngest child in the home’, ‘no fixed place to live’, characteristics of children: medically fragile/insufficient growth child’ and ‘primary caretaker has past/current mental health problems’ improved Cronbach’s alpha to .70.

### Adding experimental items to the CFRA

Table 6 shows the point-biserial correlations (*r*_pb_) and the AUC values predicting future reports of child maltreatment for: (a) the total score of the experimental items with a significant association with future reports of child maltreatment (see Table 7: item 4 and item 6-9), (b) the sum of the items of the CFRA that were significantly associated with future reports of child maltreatment (see Table 6: items 1, 2, 6 and 8d (neglect) and items 1, 2, 5a, 6, 7b, and 7c (abuse) and (c) the sum of (a) en (b)).

**Table 6:** AUC values of various sum scores in predicting future reports of child maltreatment

	AUC (95 % C.I.)
Sum of significant experimental items	.775 (.678-.873)
Sum of significant CFRA items	.750 (.631-.869)
Sum of significant CFRA items and significant experimental items	.799 (.704-.895)

**Table 7:** New set of items (CFRA and experimental items that are uniquely associated with future reports of child maltreatment)

	Item	Responses	Risk score
1.	Current intervention focuses on neglect	No	0
		Yes	1
2.	Number of prior interventions, investigations or reports (for neglect)	None	0
		One or more (general)	1
		One or more for neglect	2
3.	Current intervention focuses on abuse	No	0
		Yes	1
4.	Number of prior interventions, reports investigations (for abuse)	None	0
		One	1
		Two or more	2
4.	Prior injury to a child resulting from child abuse or neglect	No	0
		Yes	1
5.	Primary caretaker’s assessment of incident	Not applicable	0
		The fault lay with the child	1
5.	Primary caretaker provides physical care inconsistent with child’s needs	No	0
		Yes	1
6.	Primary caretaker has a current drug problem	No	0
		Yes	1
7.	Domestic violence in household in the past year	No	0
		Yes	2
8.	Primary caretaker characteristics	Overly strict with the child	1
		Very dominant	1
9.	The family has financial problems	No	0
		Yes	1
10.	Primary caretaker is involved in destructive relationships	No	0
		Yes, but not as a victim of domestic violence	1
		Yes, as a victim of domestic violence	2
9.	Activities to improve parenting skills	Performs all agreed actions	0
		Performs only some agreed actions	1
		Performs practically no agreed actions	2
		Not to be determined	1
10.	Participation to the intervention: primary caretaker cancels appointments/is not present at appointments	Never without a good reason	0
		Once	1
		More than once	2
		Not to be determined	1
11.	Primary caretaker believes that the parenting problems are less severe than indicated by the professional	No	0
		Yes	1
	Maximum points		18

The AUC value of the sum of significant CFRA items was .750, the AUC value of the sum of experimental items was .775 and the AUC value of the sum of significant CFRA items and significant experimental items was .799. The differences between these AUC values and the AUC value of the CFRA total score (.719) were not significant.

### Modified scoring procedure

**Fig. 1:**
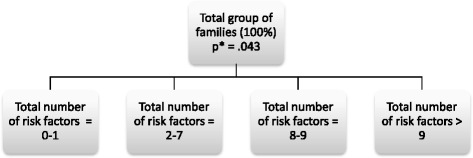
Results of CHAID analysis AUC = .795 (.694 - .895). Note * refers to the probability of future reports of child maltreatment

To examine whether the predictive validity could be further improved, a Chi-squared Automatic Interaction Detector (CHAID) analysis was performed. The items with a significant association with future reports of child maltreatment were included as independent variables for this analysis, including the sum variables (sum of (a) the significant CFRA items, sum of (b) the significant experimental items and sum of (a) and (b)). Figure 1 shows the CHAID output for the analysis.

**Fig. 2:**
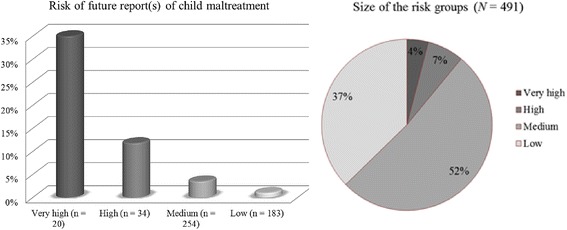
Risk of child maltreatment in the different risk groups and size of the risk groups

Future reports of child maltreatment can best be predicted by the sum of significant CFRA and experimental items. There were no other variables that added to the prediction of future reports of child maltreatment. Based on chi-squared testing, the total group was divided in four different subgroups: a group in which 0-1 risk factors are present, a group in which 2-7 risk factors are present, a group in which 8-9 risk factors are present and a group in which more than 9 risk factors are present. Figure 2 presents the risk of maltreatment in the risk groups and the size of the risk groups. The new set of items (CFRA and experimental items that are uniquely associated with future reports of child maltreatment) are presented in Table 7. Cronbach’s alpha for the new scale was .75.

### Sensitivity, specificity, false positives and negatives and Youden’s index

Table 8 shows the sensitivity, specificity and the false positives and negatives at the different cut off scores. At a cut-off score between ‘high’ and ‘moderate’ Youden’s index is highest (.433). At a cut-off score between ‘moderate’ and ‘low’, the percentage of false positives is 59 % and the percentage of false negatives is 0,2 %. At a cut-off score between ‘high’ and ‘moderate’, the percentage of false positives is 9 % and the percentage of false negatives is 2 %. At a cut-off score between ‘very high’ and ‘high’, the percentage of false positives is 3 % and the percentage of false negatives is 3 %.

**Table 8:** Sensitivity, specificity, Youden's index, false positives and false negatives for the different cut scores of the new set of items (CFRA and experimental items that are uniquely associated with future reports of child maltreatment)

Cut-off score between	Sensitivity	Specificity	Youden’s index	False positives	False negatives	Total erroneous decisions
Moderate-Low	.952	.387	.339	.587	.002	.589
High – Moderate	.524	.909	.433	.088	.020	.108
Very high – High	.333	.972	.305	.026	.029	.055

## Discussion

The aims of the present study were to examine: (a) the predictive validity of the CFRA in the Netherlands in families that received family support, (b) the added value of experimental items on the predictive validity, and (c) whether the predictive value can be improved by modifying the scoring procedure. Results showed that the predictive validity of the CFRA was modest with an AUC of .693. Adding some of the experimental items and modifying the scoring procedure by including only items that were significantly associated with future maltreatment reports resulted in a ‘high’ predictive validity (AUC = .795), however the difference between the AUC values was not significant.

In this study, it was found that CFRA items most strongly related to future reports of child maltreatment were the number of prior neglect or abuse interventions, reports and/or investigations, and domestic violence. Hindley, Ramchandani, and Jones [39] conducted a review and found similar results: They stated that three of the four most consistent risk factors for recurring maltreatment include the number of previous episodes of maltreatment, number of previous episodes of neglect, and parental conflict. Remarkably, we found that many of the individual items of the CFRA, though ostensibly referring to characteristics of dysfunctional families, were not significantly related to future reports of child maltreatment. These results are in line with the results of the validation study by Johnson [27] in which was found that only half of the CFRA items were statistically significantly related to maltreatment recurrence within two years.

There are a number of possible explanations for the finding that about half of the CFRA items were not related to child maltreatment. First, the CFRA items may relate to factors that are not very strong predictors of future child maltreatment. For example, some of the CFRA items measure the characteristics of children in the household such as delinquency, developmental disorder, intellectual disability, medical fragility and mental health problems. Results from meta-analytic reviews of the literature on risk factors for child maltreatment [34, 40] showed that most child characteristics are not related to child maltreatment. Cash [41] found that characteristics of children are not independent causal factors that increase the risk of child maltreatment, but these factors contribute to the risk of child maltreatment if other risk factors are present in the family. So the finding that CFRA items measuring characteristics of children in the household were not related to future reports of child maltreatment, both in the present study and in the study of Johnson [26], was in line with the results of earlier studies. Second, the outcome measure of the present study (future reports of child maltreatment within a period of six months) does not cover all future incidents of child maltreatment, because only the most obvious and visible incidents are reported, and a time period of six months, due to limited financial resources, is short. Third, some of the items are very difficult to assess, such as a parent’s history of abuse or neglect as a child. Therefore, these items may not be properly measured and, as a result, the associations between these items and future child maltreatment may not be properly examined.

The predictive validity of the CFRA in the Netherlands proved to be modest, with an AUC of .693. This average AUC value corresponds to a medium effect size, according to Rice and Harris [42], but is considered moderate according to generally accepted criteria, because AUC values are considered ‘acceptable’ only from .70 upwards, and ‘good’ from .75 upwards. The AUC we found in the present study is slightly higher than the AUC value found in the study by Johnson [27] of .63. The AUC value of the risk score for abuse (.719) was higher than the overall CFRA risk score. This shows that the scoring procedure of the CFRA is suboptimal for the Dutch situation, because the AUC value of the overall risk score should be at least as high as the AUC values of the risk scores for abuse and neglect. Furthermore, the predictive validity of the CFRA could be improved to an AUC of .750 by adding only the significant items instead of adding all items.

The included experimental items with a relatively strong association with future reports of child maltreatment were: ‘primary caretaker believes that the parenting problems are less severe than indicated by the professional’, ‘primary caretaker is involved in destructive relationships’, ‘the extent to which the primary caretaker performs activities to improve parenting skills’, ‘the extent to which the primary caretaker cancels appointments/is not present at appointments’ and ‘the family has financial problems’. The predictive validity of the original instrument was improved by adding these experimental items and modifying the scoring procedure by including only CFRA items that are significantly associated with future maltreatment reports (AUC = .799). This AUC value meets the generally accepted criteria for high prediction. This is an important finding since this new set of items could form a valuable instrument that is timesaving due to the limited variables included. Moreover, items that are perceived by professionals as difficult to assess, such as parent’s alcohol/drugs abuse or parents’ history of abuse/neglect, could be omitted without compromising predictive validity. However, other studies have found that these items are related to child maltreatment [34] and it might therefore be worthwhile to train professionals working with the CFRA on communication skills to talk to parents about topics that seem to be difficult for them, like the parents’ own childhood abuse or neglect.

Another advantage of this new set of items is that Cronbach’s alpha for this new scale (.75) was better than the Cronbach’s alphas of the original scales (.38 for the neglect scale, .54 for the abuse scale and .61 for the total scale) and higher than the common used lower boundary of .70. This new set of items might constitute a screening instrument with the aim of classifying children into risk groups and therefore does not have to provide a comprehensive overview of the existing problems. In the case of a high risk, children and their families should be referred for further screening with a comprehensive set of dynamic risk factors for child maltreatment so that targets of interventions can be identified and children and families can be referred to appropriate interventions.

In deciding on the precise cut-off point for referring/not referring for further assessment, both the sensitivity and specificity or both the number of false positives and false negatives should be taken into account. This is also true for the consequences of a false positive/false negative. The consequence of a false negative is that a child is not referred for further assessment, while the child is actually at risk of maltreatment, which has more serious consequences than a false positive (a child is referred for further assessment while in reality the child is not at risk of maltreatment). Of course it is important that a false positive will not lead to parents being stigmatized. To protect children as well as possible, it is important to minimize the number of false negatives. However, this is accompanied by a high number of false positives, which means that young people and their families are unnecessarily referred for further investigation. The percentage of false negatives is lowest at a cut-off score between moderate and low (0,2 %). However, at this cut-off, the percentage of false positives is 59 %. Therefore a cut-off score between high and moderate is preferable, with a percentage of false negatives of 2 % and the percentage of false positives of 8,8 %. Youden’s index is also highest at this cut-off score.

Some limitations of the study should be mentioned. Most limitations were related to the outcome measure of our study, which consisted of reports of child maltreatment at the ARCAN during a follow-up period of 6 months after completing the CFRA. Firstly, limited financial resources prevented us to verify the 6-month follow-up reports of child maltreatment by field investigation. As a result, our outcome measure might consist partly of false reports. In practice, however, most reports turn out to be correct; only 18 % of the reports to the ARCAN in the Netherlands appear to be unjustified (child maltreatment undetectable or not present). A second limitation of the outcome measure is that not every case of child maltreatment is reported to the ARCAN. The Netherlands’ Prevalence study on Maltreatment of children and youth estimated the number of reported cases of child maltreatment to the ARCAN to be around 20 % of the total number of cases of child maltreatment [1]. Unfortunately, we did not have the possibility to ask multiple resources, such as police officers, teachers, nursery teachers, and social workers at the Child Welfare Agency, about child maltreatment during the follow-up period. Therefore, our rates of child maltreatment might be an underestimation of the actual rates. Finally, some forms of maltreatment, such as emotional abuse and neglect, are less visible and therefore more difficult to detect [43]. As a result, the number of cases of emotional abuse and neglect may be underreported. In summary, a number of cases of child maltreatment are probably not detected and there may be some unfounded reports. This may have influenced the results of our study. However, our results are in line with the results of the validation study by Johnson [27] who found an AUC value of the CFRA of .63. The outcome measure of the Johnson study was substantiated by reports of child maltreatment during a follow-up period of 2 years after completing the CFRA. Another major limitation of the study is that the psychometric properties of the new set of items ought to be tested in an independent dataset to examine whether the items form a valuable risk assessment instrument, so further research on the psychometric properties is necessary. Finally, an important limitation is that the outcome of the CFRA may have influenced the behavior of the nurses, which may have influenced the AUC value. It cannot be excluded that the outcome of the CFRA has led to reports being made to the ARCAN by family coaches and intervention nurses. In addition, if the CFRA indicated a high risk, family coaches and intervention nurses probably took action to decrease the risk. Although the intervention was roughly the same for all families and the intensity of the intervention could not be adjusted based on the outcome of the CFRA, it is possible that the outcome of the CFRA has prevented individual cases of child maltreatment by a proper referral to appropriate help. If this has been the case, the predictive validity was underestimated.

## Conclusions

The results of this study are very important for detecting unsafety in child and youth care services in the Netherlands. A promising set of items has been found that could constitute a better risk assessment of child maltreatment. In addition, this new set of items saves time because only variables that uniquely contribute to the prediction of future reports of child maltreatment are included. The aim of the new set of items is to classify children into risk groups (first screening of risk). In the case of a high risk, children and their families should be referred for further screening with a comprehensive set of dynamic risk factors for child maltreatment so that targets of interventions can be identified and children and families can be referred to appropriate interventions. Further research is needed to confirm the good psychometric properties of this new set of items and its value for the prediction of child maltreatment (cross validation). This is important because actuarial methods (simple risk assessment procedures with known predictive attributes) are of great value in the context of increased demands placed on child protection services in an era of declining resources.

## Additional file

Additional file 1:
**Items of the CFRA, respons options, and risk scores.** (DOCX 18 kb)
