# Supplementary material for: Detection of unsafety in families with parental and/or child developmental problems at the start of family support
Source: BMC Psychiatry. 2016 Jan 21;16:15. doi: 10.1186/s12888-016-0715-y (PMC4722745; doi:10.1186/s12888-016-0715-y)
Supplement: Additional file 1: — Items of the CFRA, respons options, and risk scores. (DOCX 18 kb) [file 12888_2016_715_MOESM1_ESM.docx]

**Additional file**

*Items of the CFRA assessing the likelihood of future neglect.*

|  | Item | Responses | Risk score |
| --- | --- | --- | --- |
| 1. | Current intervention is aimed at neglect | No  Yes | 0  1 |
| 2. | Number of prior interventions, investigations or reports (for neglect) | None  One or more (general)  One or more for neglect | 0  1  2 |
| 3. | Household has previously received child protection services (voluntary/court-order) | No  Yes | 0  1 |
| 4. | Number of children involved in child abuse/neglect incidents | 1-3  4 or more | 0  1 |
| 5. | Age of youngest child in the home | Two years or older  Younger than two years | 0  1 |
| 6. | Primary caretaker provides physical care inconsistent with child’s needs | No  Yes | 0  1 |
| 7. | Primary caretaker has a past or current mental health problem | No  Yes | 0  1 |
| 8. | Primary caretaker has a historic or current alcohol or drug problem | No  Yes | 0  1 |
| 9. | Characteristics of children in the household | Not applicable  Medically fragile/ insufficient growth  Developmental disorder or disabled  Intoxicated at birth | 0  1  1  1 |
| 10. | Housing | No problems  Current housing is unsafe  No fixed place to live | 0  1  2 |
|  | Maximum points |  | 18 |

*Items of the CFRA assessing the likelihood of future abuse (both physical and sexual)*

|  | Item | Responses | Risk score |
| --- | --- | --- | --- |
| 1. | Current intervention is aimed at abuse | No  Yes | 0  1 |
| 2. | Number of prior interventions, reports investigations (for abuse) | None  One  Two or more | 0  1  2 |
| 3. | Household has previously received child protection services (voluntary/court-order) | No  Yes | 0  1 |
| 4. | Prior injury to a child resulting from child abuse or neglect | No  Yes | 0  1 |
| 5. | Primary caretaker’s assessment of incident | Not applicable  The fault lay with the child  The caretaker justified the abuse | 0  1  2 |
| 6. | Domestic violence in the household in the past year | No  Yes | 0  2 |
| 7. | Primary caretaker characteristics | No problems  Insufficient emotion and psychological support  Overly strict with the child  Very dominant | 0  1 |
| 8. | Primary caretaker has a history of abuse or neglect as a child | No  Yes | 0  1 |
| 9. | Secondary caretaker has historic or current alcohol or drug problem | Not applicable  Yes | 0  1 |
| 10. | Characteristics of children in the household | No problems  Delinquency  Developmental disorder /intellectual disability  Mental health problems or behavioral problems | 0  1  1  1 |
|  | Maximum points |  | 18 |

*Scoring of the CFRA*

| Score Neglect | Score Abuse | Risk level |
| --- | --- | --- |
| 0-1 | 0-1 | Low |
| 2-4 | 2-4 | Moderate |
| 5-8 | 5-7 | High |
| 9 or more | 8 or more | Very high |
